# Clinicopathological Features and Anatomical Patterns of Head and Neck Tumors From Cape Coast Teaching Hospital and Komfo Anokye Teaching Hospital in Ghana: A Cross‐Sectional Study

**DOI:** 10.1002/hsr2.72395

**Published:** 2026-04-23

**Authors:** Roland Osei Saahene, Precious Barnes, F. A. Yeboah, Du‐Bois Asante, Elvis Agbo, Samuel Kofi Arhin, Sylvester Ackah Famieh

**Affiliations:** ^1^ Department of Microbiology and Immunology, School of Medical Sciences, College of Health and Allied Sciences University of Cape Coast Cape Coast Ghana; ^2^ Department of Chemical Pathology, School of Medical Sciences, College of Health and Allied Sciences University of Cape Coast Cape Coast Ghana; ^3^ Department of Molecular Medicine, School of Medical Sciences, College of Health Sciences Kwame Nkrumah University of Science and Technology Kumasi Ghana; ^4^ Department of Forensic Sciences, School of Biological Sciences, College of Agricultural and Natural Sciences University of Cape Coast Cape Coast Ghana; ^5^ Department of Human Anatomy, Histology and Embryology, College of Medicine Jinggangshan University Ji'an City China; ^6^ Department of Physiology, School of Medical Sciences, College of Health and Allied Sciences University of Cape Coast Cape Coast Ghana; ^7^ Department of Physician Assistant Studies, School of Medical Sciences, College of Health and Allied Sciences University of Cape Coast Cape Coast Ghana

**Keywords:** head and neck tumor, mandible, oral cavity

## Abstract

**Background:**

There is still a dearth of information on the prevalence and histological patterns of head and neck tumors (HNT) in Ghana, which emphasizes the need for regional studies to learn more about their distribution and clinical features.

**Aim:**

Thus, the present study aimed to describe the anatomical pattern and clinicopathological features among patients with HNT at some selected hospitals in Ghana.

**Method:**

The study was retrospective in design, which consisted of laboratory analysis of archived HNT tissues. The study was conducted with 250 archived paraffin‐embedded HNT tissue samples from the Pathology Department of Cape Coast Teaching Hospital and Komfo Anokye Teaching Hospital in Ghana. The various tumor grades were analyzed using hematoxylin and eosin staining on tissue samples from a consecutive series of 150 HNT samples, out of 250 patients who underwent tumor resection over a 4‐year period.

**Results:**

There were 148 males and 102 females, giving a male‐to‐female ratio of 1.45:1. The age range was 10–89 years. The commonest anatomical site of patients with the HNT was the oral cavity (34%), followed by the nasal cavity (25.2%) and mandible (24%). The majority of the patients with HNT were recorded in 2018 (23%).

**Conclusion:**

This study reveals that the oral cavity is the commonest HNT in Ghana, with a higher occurrence observed in patients above 40 years old. Therefore, there is a need to undertake public health education to raise awareness to promote early detection and management of the disease.

## 1 Introduction

Head and neck tumors (HNT) are tumors that occur in the mucosal linings of the upper aerodigestive tract, which include the oral cavity, nasal cavity, paranasal sinuses, pharynx, and larynx. Clinically, it is considered a heterogeneous disease with the majority of the cases presenting as squamous cell carcinomas [1, 2]. High consumption of tobacco and alcohol causes the aetio‐pathogenesis of the tumor as well as high‐risk human papillomavirus (HPV) subtypes [2, 3, 4].

Variations in the alcohol dehydrogenase 1B (ADH1B) and aldehyde dehydrogenase 2 (ALDH2) genes have been strongly associated with alcohol intake and HNT [5]. Studies concentrating on particular anatomical subsites of head and neck malignancies are noticeably lacking in Ghana. The research currently in publication frequently lumps these malignancies together without making a distinction between the several subsites, including the salivary glands, pharynx, larynx, nasal cavity, and oral cavity. Furthermore, there is still a dearth of thorough information regarding the histological characteristics, anatomical distribution, and occurrence of head and neck tumors. Strategies for focused prevention, early detection, and treatment that are suited to the Ghanaian population are hampered by this gap in epidemiological and clinical expertise.

Head and neck tumors are the sixth most common tumors and also result in significant morbidity [6]. Symptoms of the tumor include difficulty in swallowing, numbness in the neck, leukoplakia, erythroplakias lesions around the mouth. It can occur in different anatomical sites [7]; hence, its anatomical patterns must be clearly understood to enhance effective treatment of the disease to aid in improving survival outcomes. The present study, therefore, sought to investigate the anatomical pattern of head and neck cancers in Ghana and their correlation with clinicopathological parameters.

## 2 Methods

The study protocol was retrospective in design, which consisted of laboratory analysis of archived HNT tissue samples. Tissue samples were obtained from a total of 250 archival head and neck tumor tissues. The HNT tissue samples used in this investigation were archival formalin‐fixed, paraffin‐embedded (FFPE) samples obtained from the pathology departments of the Cape Coast Teaching Hospital (CCTH) and Komfo Anokye Teaching Hospital (KATH) in Ghana. Histologically verified primary head and neck tumor specimens diagnosed between 2014 and 2018 were among the eligible cases. 250 samples with sufficient tissue preservation and comprehensive clinical and demographic information, including age, gender, and tumor site for the overall generala characteristics.

Any cases with significantly deteriorated tissue blocks or missing critical clinical information, were not included. To preserve patient privacy, all samples were anonymized prior before analysis.

### 2.1 Clarification on Sample Selection

Of the 250 archival HNT tissue samples initially retrieved, only 150 were ultimately included in the study for Hematoxylin and Eosin (H&E). This selection was based on the quality and completeness of H&E staining. All samples were subjected to standard FFPE tissue sectioning and stained using routine H&E protocols, with commercial control slides employed to ensure staining consistency and reliability.

However, upon microscopic evaluation, 100 of the stained tissue sections were excluded due to inadequate tissue preservation, incomplete staining, or insufficient tumor content that did not meet the criteria for accurate histopathological assessment. Therefore, only 150 samples with interpretable and diagnostically adequate H&E staining results were used for subsequent analyses. This quality control step was essential to maintain the integrity and validity of the histological assessments included in the study.

Both benign and malignant head and neck tumors were classified according to the current World Health Organization's International Classification of Diseases coding system [8].

### 2.2 Ethical Consideration

Prior to the initiation of this study, the research and the project protocol were approved by Ethical Review Committee of the Cape Coast Teaching Hospital and the Committee on Human research, publications (CCTHERC/RS/EC/2017/52) and Ethics of Kwame Nkrumah University of Science and Technology, School of Medical Sciences and Komfo Anokye Teaching Hospital (CHRPE/RC/149/18).

### 2.3 Statistical Analysis

Stata/SE version 11.1 (StataCorp, College Station, TX, USA) was used for all statistical analyses. Frequencies and percentages were used to summarize categorical variables. The *χ*² test was used to evaluate relationships between categorical variables, such as tumor grade or stage and gender. Fisher's exact test was used to make sure the results were valid in situations when the expected cell frequencies were less than five.

Stratified subgroup analyses were carried out independently for male and female patients to investigate whether relationships differed by gender. Odds ratios (ORs) with associated 95% confidence intervals (CIs) were used to quantify the direction and degree of correlations. A *p* value of less than 0.05 was deemed statistically significant, and all statistical tests were two‐sided.

Every statistical symbol, word, and acronym used in the analysis—such as OR, CI, and *χ*²—is defined when it first appears in the text. Unless otherwise indicated, no exploratory analyses were carried out; the analyses mentioned above were pre‐specified before data collection.

## 3 Results

**Figure 1:**
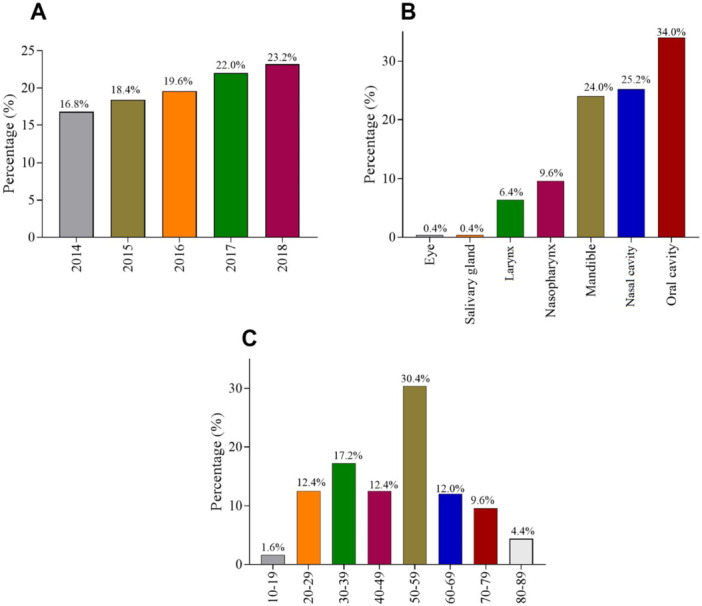
Annual occurrence, age, and anatomical site of the head and neck tumor.

A total of 250 tissue archival HNT samples were studied. Most of the samples were cases from the year 2018 (23.2%), followed by 2017 (22.0%) (Figure 1A). The highest proportion of samples per anatomical site across the 4‐years were oral cavity (34.0%), followed by nasal cavity (25.2%), and mandible (24.0%) (Figure 1B). The average age of the patients was 49.7 years, with the majority of them being between the ages of 50–59 years (30.4%) (Figure 1C).

Of the 250 tumors confirmed to be head and neck tumors by WHO criteria, 148 and 102 were from male and female patients, respectively. The male‐to‐female ratio was 1.45:1. Among the males, the anatomical site with the highest head and neck tumor was observed for the oral cavity (39.2%), followed by the nasal cavity (29.7%). In the females, mandible tumor was the highest occurring tumor (28.4%) followed by tumor of the oral cavity (26.5%) (Table 1).

**Table 1:** General characteristics of the studied population stratified by gender.

Variables	Total (*n* = 250)	Female (*n* = 102)	Male (*n* = 148)
Age (years)			
≤ 40	83 (33.2)	39 (38.2)	44 (29.7)
> 40	167 (66.8)	60 (58.8)	107 (72.3)
Year			
2014	42 (16.8)	17 (16.7)	25 (16.9)
2015	46 (18.4)	18 (17.6)	28 (18.9)
2016	49 (19.6)	22 (21.6)	27 (18.2)
2017	55 (22.0)	23 (22.5)	32 (21.6)
2018	58 (23.2)	22 (21.6)	36 (24.3)
Tumor site			
Eye	1 (0.4)	0 (0.0)	1 (0.7)
Mandible	60 (24.0)	29 (28.4)	31 (20.9)
Oral cavity	85 (34.0)	27 (26.5)	58 (39.2)
Nasal cavity	63 (25.2)	19 (18.6)	44 (29.7)
Nasopharynx	24 (9.6)	8 (7.8)	16 (10.8)
Salivary gland	1 (0.4)	0 (0.0)	1 (0.7)
Larynx	16 (6.4)	5 (4.9)	11 (7.4)

When tumor location was stratified by age group, one patient with an eye tumor and another with a salivary gland tumor were between the ages of 30 and 39 and 60 and 69, respectively.

The highest proportion of patients with tumors of the mandible, oral cavity, nasal cavity, nasopharynx, and larynx were within the age range of 30–39 years (26.7%), 50–59 years (20.0% vs. 39.7% vs. 50.0% vs. 43.8%), respectively (Table 2).

**Table 2:** Anatomical site distribution of head and neck tumors stratified by age ranges.

Age (years)	Eye	Mandible	Oral cavity	Nasal cavity	Naso pharynx	Salivary gland	Larynx
10–19	0 (0.0)	0 (0.0)	2 (2.4)	2 (3.2)	0 (0.0)	0 (0.0)	0 (0.0)
20–29	0 (0.0)	15 (25.0)	12 (14.1)	0 (0.0)	2 (8.3)	0 (0.0)	2 (12.5)
30–39	0 (0.0)	16 (26.7)	14 (16.5)	6 (9.5)	6 (25.0)	0 (0.0)	1 (6.3)
40–49	0 (0.0)	13 (21.7)	8 (9.4)	8 (12.7)	2 (8.3)	0 (0.0)	0 (0.0)
50–59	1 (100.0)	14 (23.3)	17 (20.0)	25 (39.7)	12 (50.0)	0 (0.0)	7 (43.8)
60–69	0 (0.0)	2 (3.3)	15 (17.6)	8 (12.7)	2 (8.3)	1 (100.0)	2 (12.5)
70–79	0 (0.0)	0 (0.0)	14 (16.5)	6 (9.5)	0 (0.0)	0 (0.0)	4 (25.0)
80–89	0 (0.0)	0 (0.0)	3 (3.5)	8 (12.7)	0 (0.0)	0 (0.0)	0 (0.0)
Total	1 (0.4)	60 (20)	85 (34.0)	63 (25.2)	24 (9.6)	1 (0.4)	16 (6.4)

**Figure 2:**
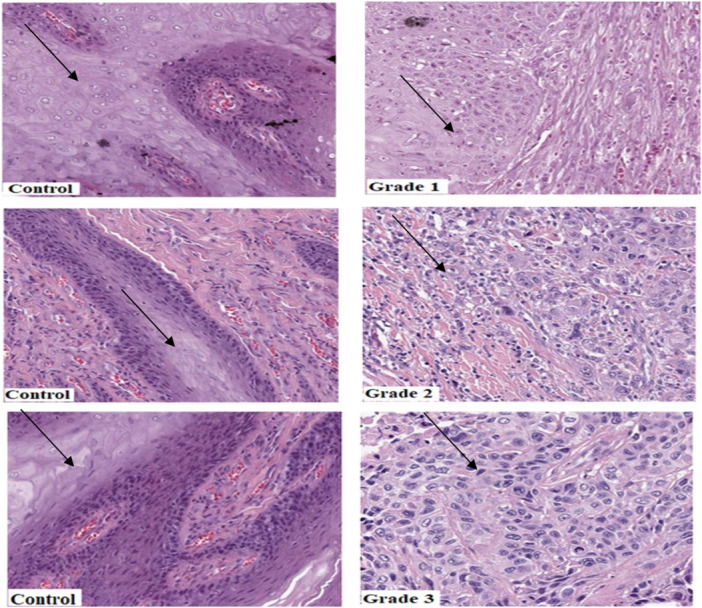
Comparison of H&E‐stained sections for tumor grade (×400). Arrows showing pool keratin pearls for control and Grade 1 tumor. Grade 2; an arrow showing individual cells' keratinization. Grade 3; no keratinization forming in the tumor cells.

Histological differentiation was conducted with 150 tissues of the study population and they were compared with commercial positive controls. The proportions of well, moderate and poorly differentiated malignant tissues were 32.7%, 50%, and 17.3%, respectively. *Chi‐squared* test of independence revealed a statistically significant association between gender and tumor differentiation (*p* = 0.003) (Table 3). Figure 2 shows the comparison of H&E‐stained sections with respect to tumor grade. Grade 1 cells were similar to those of the control and there was a pool of keratin pearls similar to that of the control cells. For the Grade 2, the cells were slightly differentiated from the control cells and there was individual cell keratinization. The Grade 3 cells were differentiated from the control cells with no keratin present.

**Table 3:** Stratification of gender based on tumor grade and stage.

	Total (*n* = 150)	Female (*n* = 60)	Male (*n* = 90)	*χ* ^2^	*p* value
Grade					
Well	49 (32.7)	14 (23.3)	35 (38.9)	8.930	0.003
Moderate	75 (50.0)	42 (70.0)	33 (36.7)		
Poor	26 (17.3)	4 (6.7)	22 (24.4)		
Stage					
I/II	51 (42.4)	17 (63.0)	34 (47.9)	1	
III/IV	47 (48.0)	11 (37.0)	36 (52.1)	1.64 (0.67–3.99)	0.279

## 4 Discussion

HNT are prevalent among old people, especially in HPV‐negative persons [9, 10]. From the current study, the incidence of these tumors was low among persons younger than 40 years. Most of the patients (66.8%) in our study were aged above 40, with 33.2% being younger than 40 years (Table 1). This finding corroborates a study by Bhattacharjee et al. [11] who reported a prevalence rate of 54.48% in persons between 40 and 69 years.

Out of a total of 250 patients, 148 were men, and 102 were women (Table 1). The male‐to‐female ratio was 1.45:1 with a mean age of 49.7 years (range, 18–89 years). This is consistent with a study by Larsen‐Reindorf et al. at Komfo Anokye Teaching Hospital in Ghana [12], who found the same ratio but with a mean age of 48 years. Head and neck tumors are more commonly seen in males due to high prevalence of smoking and alcohol consumption among men [13]. The lower prevalence of tobacco and alcohol‐related tumors among women could be a result of the low usage of tobacco and alcohol among Ghanaian women [14].

The oral cavity was found to be the highest anatomical site for the HNT comprising of 34.0% (Table 1). This is in consonance with a study by Abdul‐Hamid et al. [15], who reported that oral cavity tumors were the most common, with a prevalence of 31.7% in their study of 183 patients with head and neck cancers. Similar findings have also been reported [10, 11]. From this study, the prevalence of the oral cavity was followed by the tumor of the nasal cavity (25.2%), the mandible (24%), and the least anatomical site seen in both the salivary gland (0.4%) and the eye (0.4) (Table 1).

Tumors in the oral cavity are mostly the result of smoking and alcohol. Smoking and alcohol increase the risk of oral cavity by two or threefold [6]. Though this study did not focus on the etiology of the cases reported, the consumption of both alcohol and tobacco has been linked to a higher incidence than tobacco or alcohol alone [16]. The first site of food into the body is the oral cavity. It is also the site where exogenous materials such as microorganisms and harmful agents, are high. The immunological barrier in the oral cavity is the oral mucosa. Hence, it prevents the infections from parasites, fungi, bacteria, viruses, and also carcinogens such as aromatic hydrocarbons and nicotine [17].

Patients with tumors in the oral cavity usually have leukoplakia, which is a protective mechanism against exogenous materials. It involves an increase in the epithelial formation and high keratinization, which is presented as a white discoloration of the mucosa [18]. The protective mechanism can be reversible, but when the mechanism is not controlled, tumorigenesis is favored. Endogenous factors such as genetic alteration, hormonal factors, hepatic disease, and other viral infections also influence these changes [19, 20, 21].

Morphological assessment of tumors was classified based on the cell differentiation into well, moderately, poorly differentiated carcinomas, and anaplastic tumors. Grading was also based on the amount of keratinizing islands and how the tumor cells had been differentiated from the normal cells. Poorly differentiated head and neck tumor cells normally spread from their original site through the regional lymph nodes after invading the connective tissue and muscle [22]. Poorly graded cells or immature cells are abnormal cells and are more aggressive in cell division [23]. The poorly differentiated cells, which are the high‐grade, are more aggressive and tend to metastasize to regional lymph nodes even at an early stage of the tumorigenesis. From the current study, it was observed that the Grade 1 tumor presented with prominent keratin pearls and intercellular bridges. The cells were also well‐differentiated and were similar to those of the normal cells. Grade 2 tumors were observed to be moderately differentiated with few keratin pearls and poorly defined intercellular bridges. Grade 3 tumors showed poorly differentiated cells with no keratin, and frequent mitotic figures (Figure 2). The loss of keratinization and the loss of intercellular bridges made the tissue more compact. This shows that the tumor cells were actively dividing. This observation is in accordance with a study done by Cohen and Wenig [24].

Cell division is an important factor in maintaining tissue integrity. In cancer, abnormal cell growth and cell division result in excessive cellular proliferation. Dysplasia is associated with altered tissue architecture, including cellular proliferation leading to the malignant transformation if left untreated [25].

In the present study, all the participants were diagnosed with squamous cell tumors. The majority of the tumors were moderately differentiated tumors, representing 50% of the studied population, followed by the well‐differentiated tumors 32.7% and poorly differentiated tumors being the least, accounting for 17.3% (Table 3). A similar finding was observed in a study conducted on 198 patients in which 160 (81%) were moderately differentiated squamous cell carcinoma, 33 (16.5%) were well‐differentiated, and 5 cases (2.5%) were poorly differentiated squamous cell tumors [25]. Hence, treatment strategies should focus more on moderately differentiated squamous cell carcinoma to improve survival rates among affected individuals. In prognostic approaches, individuals undergoing treatment should also be cautioned of the risk of reemergence of the cancer. This is because continual alcohol consumption and smoking of tobacco can potentiate the development of cancer [26, 27, 28].

## 5 Conclusions

This study revealed that the oral cavity was the common anatomical site for the development of head and neck tumors, with the highest occurrence observed in patients above 40 years old among Ghanaians. Therefore, there is a need to undertake public health education to raise awareness and promote early detection and reporting to health institutions for further management.

## 6 Future Research Directions

The results of this study demonstrate significant correlations between patient demographics, including gender, and tumor features in head and neck tumors. We hope to have another study which will include information about prognostication and tailored therapy from a longitudinal study to evaluate treatment responses and survival outcomes. Furthermore, investigating lifestyle, viral (e.g., HPV, EBV), and environmental risk factors in conjunction with genetic changes may help us better understand the pathophysiology of HNT in the Ghanaian community.

## Author Contributions

Roland Osei Saahene and Precious Barnes conceived and designed the study. F. A. Yeboah, Elvis Agbo, and Samuel Kofi Arhin collected clinical data and samples. Du‐Bois Asante and Precious Barnes performed laboratory analyses. Roland Osei Saahene, Sylvester Ackah Famieh, and Samuel Kofi Arhin analyzed the data.

## Funding

The authors have nothing to report.

## Conflicts of Interest

The authors declare no conflicts of interest.

## Transparency Statement

The corresponding author, Precious Barnes, affirms that this manuscript is an honest, accurate, and transparent account of the study being reported; that no important aspects of the study have been omitted; and that any discrepancies from the study as planned (and, if relevant, registered) have been explained.

## Data Availability

Since the data contains sensitive identifying information, it cannot be publicly disseminated. For researchers who meet the requirements for access to confidential data, however, information is available from the Cape Coast Teaching Hospital Ethical Review Committee (CCTHERC), the ethics board of the Kwame Nkrumah University of Science and Technology's School of Medical Sciences, KNUST, and Komfo Anokye Teaching Hospital (KATH), Kumasi, Ghana (email: cctherc@gmail.com, chrpe.knust.kath@gmail.com).
